# Lymphocytic gastritis in a patient with dyspepsia

**DOI:** 10.1002/ccr3.2308

**Published:** 2019-07-21

**Authors:** Katrina Collins, William N. Rezuke

**Affiliations:** ^1^ Department of Pathology Hartford Hospital Hartford Connecticut

**Keywords:** celiac disease, *Helicobacter pylori*, intraepithelial lymphocytosis, lymphocytic gastritis, lymphocytic gastroenterocolitis, MALT lymphoma

## Abstract

Lymphocytic gastritis (LG) is uncommon and presents histologically with a nonspecific inflammatory pattern. It is most often associated with celiac disease and *Helicobacter pylori* gastritis and is rarely associated with other conditions including lymphoma. LG is of clinical importance since its recognition should prompt further clinical evaluation for other disorders.

Lymphocytic gastritis (LG) is associated with celiac disease and *Helicobacter pylori* infection, and rarely with other conditions including lymphoma.^1^

A 50‐year‐old female presented with a 2‐week history of epigastric pain, nausea, vomiting, and weight loss. An abdominal CT scan and endoscopy were unremarkable. A gastric biopsy was obtained.

The biopsy revealed a moderately dense lymphocytic infiltrate involving the lamina propria. In addition, the surface epithelial cells and gastric pit demonstrated markedly increased intraepithelial lymphocytes (IELs) with >25 IELs/100 epithelial cells. The IELs were predominantly small lymphocytes, including many with a halo appearance (Figure 1). The intraepithelial infiltrate raised concern for extranodal marginal zone lymphoma (MALT lymphoma).

By immunohistochemistry, the lymphoid infiltrate consisted of mostly CD3+/CD8+/TIA‐1 + cytotoxic T lymphocytes, characteristic of LG. CD4+ T cells localized to the lamina propria without epithelial involvement (Figure 1). An immunostain for *H pylori* was negative. In contrast, MALT lymphoma is characterized by a monoclonal B‐cell infiltrate. Follow‐up serologic studies for celiac disease and *H pylori* were negative.

**Figure 1:**
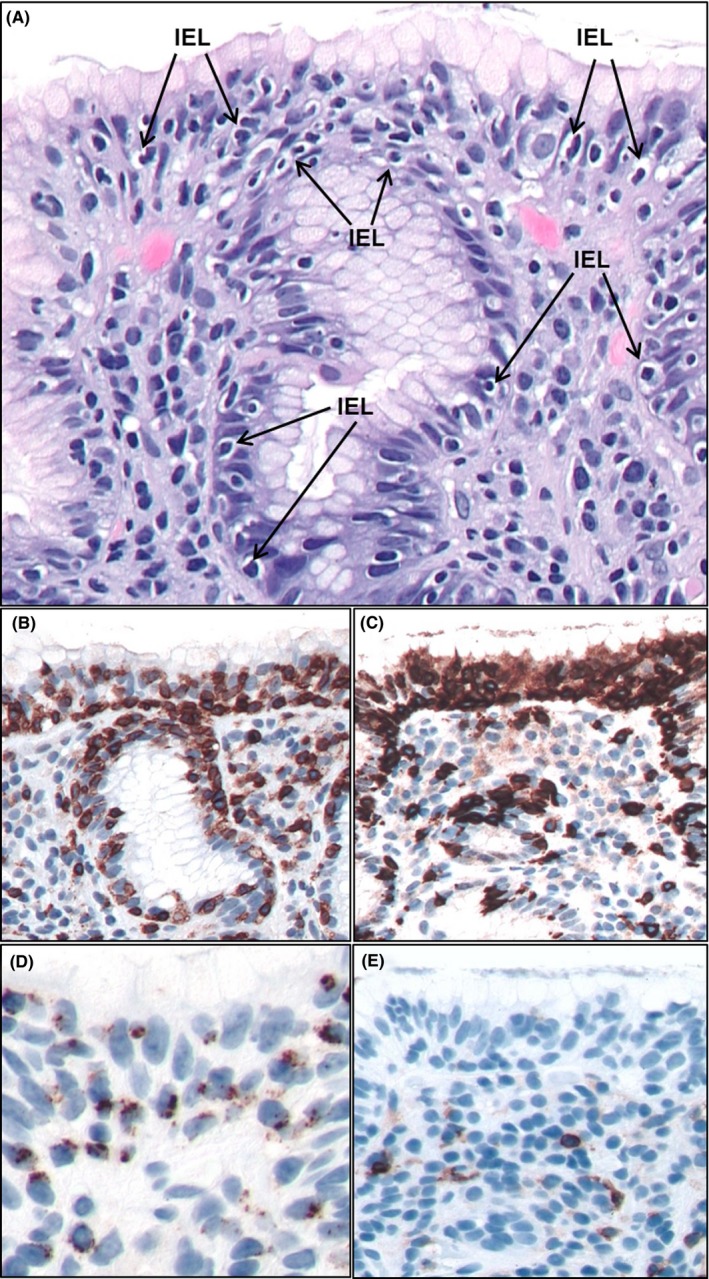
A hematoxylin‐and‐eosin–stained section of the gastric biopsy demonstrates surface epithelium and gastric pit, with numerous intraepithelial lymphocytes (arrows) including many lymphocytes with a halo appearance (A). By immunohistochemistry, the IELs were positive for CD3 (B), CD8 (C), and TIA‐1 (D), characteristic of cytotoxic T cells and consistent with LG. CD4+ T cells (E) localized primarily to the lamina propria without significant epithelial involvement (×50 magnification). IEL, intraepithelial lymphocyte; LG, lymphocytic gastritis

A diagnosis of LG should prompt further clinical evaluation for celiac disease (including serologic testing and possible additional small bowel biopsies). Antibiotic therapy for *H pylori* infection should also be considered even if *H pylori* is not detected histologically.^2^

## AUTHOR CONTRIBUTIONS

KC: served as the primary author and is responsible for this literature review and construction of the manuscript. WNR: served as the hematopathologist on the case and was responsible for the histopathological work‐up and final diagnosis as well as senior author managing the construction and edits of the manuscript and guiding the primary author through the submission process. 
